# Promoting awareness of open access publishing in speech-language therapy and audiology: Lessons from multilingual language assessment research

**DOI:** 10.4102/sajcd.v73i1.1150

**Published:** 2026-03-31

**Authors:** Juan Bornman, Mellissa Bortz, Anita Edwards, Brenda Louw, Jeannie van der Linde

**Affiliations:** 1 Division of Speech-Language and Hearing Therapy, Faculty of Medicine and Health Sciences, Stellenbosch University, Stellenbosch, South Africa; 2 Department of Communication Sciences and Disorders, St John’s College of Liberal Arts, St John’s University, New York, United States of America; 3 Department of Speech-Language Pathology and Audiology, Faculty of Humanities, University of Pretoria, Pretoria, South Africa; 4 School of Health Sciences (Audiology), College of Health Sciences, University of KwaZulu-Natal, Durban, South Africa; 5 Department of Audiology and Speech-Language Pathology, College of Health Sciences, East Tennessee State University, Johnson City, United States

**Keywords:** open access publishing, evidence-based practice, speech-language therapy and audiology, transformative, clinician-researcher, knowledge

## Abstract

Access to speech-language therapy (SLT) and audiology (AUD) research is critical for clinicians, researchers, policymakers and the public to fulfil the professional scope of practice, support advocacy and enable evidence-based service delivery. However, research locked behind paywalls disproportionately limits access for underserved communities and for clinician–researchers seeking tools in languages with scarce resources. Limited access to clinically applicable research disproportionately affects individuals needing SLT and/or AUD services in under-resourced languages and populations, where contextualised, high-quality evidence is most needed. The authors advocate for open access (OA) publishing as a critical strategy to improve access to research in SLT and AUD. Open access publishing promotes more equitable dissemination of information, potentially fostering inclusion, innovation and informed clinical decision-making. This perspective calls for the clinician–researcher to become aware of the different OA models and how to access their benefits. Furthermore, we encourage advocacy amongst key stakeholders, highlighting how collaborative efforts may strengthen the move towards increased OA publishing.

**Contribution:**

This article contributes to the growing dialogue on decolonising knowledge and democratising access within the health sciences. By promoting OA publishing, the fields of SLT and AUD can help dismantle structural inequities in knowledge dissemination, support local and global clinical relevance and improve the quality of service for multilingual and underserved communities worldwide. We specifically call on South African clinician-researchers to engage with OA opportunities and collaborate in sharing clinicallyapplicable findings, including implementation guidance, outcome evidence, and contextual considerations for assessment and intervention in South African languages.

## Introduction

Access to research in speech-language therapy (SLT) and audiology (AUD) is critical for clinicians, researchers, policymakers, and the public to fulfil professional scope-of-practice requirements (HPCSA, 2017) and support evidence-based practice (American Speech-Language-Hearing Association [ASHA], 2005). Beyond access alone, research impact is closely tied to clinical uptake, understood as the extent to which research findings are interpreted, adopted and applied by clinicians in routine assessment, intervention and service-delivery options. Clinical uptake is, in turn, strongly influenced by the accessibility of research outputs, particularly in contexts where the burden of need is high (Trouwloon et al., 2024). In South Africa, a low- and middle-income country (LMIC) characterised by marked disparities in resources, limited accessibility to research remains a significant barrier for both researchers and clinicians (Budlender, 2018).

Our positionality is situated within academic settings, with all authors currently working as academics in the fields of SLT and AUD. Though our primary appointments are within universities, our research agendas are strongly informed by clinical practice and by ongoing engagement with clinicians, clinical training contexts and service-delivery realities in South Africa. This positioning influences both our awareness of, and access to, academic publishing systems, including the financial and structural implications of open access (OA) publishing. At the same time, it shapes our commitment to ensuring that research outputs are accessible, affordable and usable for clinicians working in diverse and resource-constrained contexts. In this paper, we therefore position clinicians – particularly those with limited access to paywalled literature – as the primary audience, with academics serving as collaborators and enablers in improving access to and the usability of research outputs.

We have written this paper as a clinical perspective opinion piece, informed by our direct experience of the challenges clinicians face in accessing relevant, usable and contextually appropriate evidence to support SLT and AUD practice in South Africa. The challenges were encountered through our shared commitment to ensuring that research results are not only generated, but are also accessible and meaningful for clinical application. In particular, we reflect on the availability of easily accessible, culturally and linguistically responsive assessment and intervention resources and consider how OA publishing can strengthen clinical uptake, local relevance and the ongoing development of E^3^BP. The E^3^BP framework, first introduced in SLT and AUD by Dollaghan (2007), provides a useful lens for this discussion. In her model, clinical expertise is informed by three sources of evidence: (E1) external evidence, (E2) internal evidence and (E3) client preferences. By integrating these elements, E^3^BP supports care that is both evidence-based and patient-centred (Ginsberg & DeRuiter, 2024), highlighting the importance of accessible and contextually relevant resources for effective practice.

The authors therefore suggest that one avenue to address these challenges is to foreground the needs of clinicians – particularly those practising in resource-constrained settings – who often have limited institutional support and restricted access to paywalled literature. Researchers and academics in the fields of SLT and AUD, specifically those working in language assessment and intervention, are encouraged to collaborate with clinicians practising E^3^BP to improve the availability of research findings and the accessibility of clinical tools. To support this aim, this opinion paper advocates increased, more strategic engagement with OA publishing as a means of addressing inequities in access to evidence. Specifically, we outline key OA models relevant to SLT and AUD, discuss their applicability in South Africa and provide practical guidance for clinician–researchers on contributing to OA publications, including funding strategies, as well as accessing research outputs that are not openly available. In acknowledging the structural barriers clinicians face outside academic institutions, we propose feasible facilitators and strategies to mitigate these challenges, with the ultimate aim of strengthening clinical impact in resource-constrained contexts.

## Open access publishing

Unlike the traditional ‘pay to read’ model, where readers or institutions must pay for access to published research, the OA ‘pay to publish’ model shifts the cost to the author. In this approach, articles are freely available to anyone, removing financial barriers for readers.

In the OA model, authors typically pay an article processing charge (APC), which varies depending on the journal and the specific open access option selected. In this context, OA refers to the free, immediate, online availability of research outputs, such as peer-reviewed journal articles and scholarly books and the rights to use these outputs in the digital environment. The OA content is open to all, with no access fees or subscription barriers (Springer Nature, 2026).

Making research open (in other words, available) and as accessible as possible increases its visibility, accelerates its impact and enhances its ability to address global challenges (ASHA, n.d.; Cambridge University Press, n.d.; Long et al., 2023).

There are several types of OA publishing models. Gold OA refers to articles that are made freely available immediately upon publication in fully OA journals, often requiring authors to pay an APC. Green OA allows researchers to make their work accessible by self-archiving a version of their articles, such as a preprint or post-print, in an institutional or subject repository. Diamond or Platinum OA journals provide OA without charging authors any fees, making them free for both readers and contributors. Lastly, Bronze OA refers to articles that are freely available to read on the publisher’s website but lack an open license, which limits how the material can be reused.

International research funders have played a pivotal role in promoting OA in scientific outputs. Their motivation is to ensure that the research they support, including data, software and other materials, is openly and easily accessible. This approach is intended to maximise the potential impact of the research and foster further innovation and collaboration. For example, the Wellcome Trust, a British charitable foundation supporting global health research, mandates that all research it funds is openly accessible to the public. Publishers must publish these articles on PubMed Central, update them if needed, use a Creative Commons license (CC BY) and offer refunds for article fees as part of their agreement with the Wellcome Trust. Other major charities, such as Cancer Research UK and the British Heart Foundation, have also agreed to follow the same rules for their funded research. Similarly, the Bill and Melinda Gates Foundation requires that the research it funds be openly accessible (and hence available) to ensure broad dissemination and impact. However, paying article fees remains challenging for researchers, especially for clinicians, if their research is not funded by the respective funders.

An example of the developments in the OA landscape is the ‘Bibliotekssamverkan’ (the BIBSAM Consortium), a Swedish national consortium coordinated by the National Library of Sweden. Translating to ‘Library Collaboration’, the consortium comprises 95 Swedish institutions. Its aim was to redirect payment streams away from subscription-based models towards OA publishing, promote transparency and reduce scholarly publishing costs for Swedish researchers. This has been a successful endeavour. The National Swedish Library has negotiated major licensing and publishing agreements with leading academic publishers (e.g. Elsevier) on behalf of Swedish universities, research institutes and public agencies (Widding, 2025).

In South Africa, the shift towards OA is gaining traction. Initiatives such as the South African National Library and Information Consortium (SANLiC), which negotiates OA agreements with publishers, have significantly contributed to this shift. The South African National Library and Information Consortium’s agreements with publishers such as Emerald, Sage, Wiley, Taylor and Francis and Springer Nature enable South African researchers to publish in these journals with either full APC waivers or significant discounts. ‘*Read and publish agreements*’ (commonly known as transformational agreements (TAs) in South Africa) play a key role in the transition to OA by covering APCs that might otherwise limit researchers’ ability to publish in open formats.

Universities and research institutions are increasingly supporting OA publishing by offering guidance and funding through their libraries and research offices. The Scientific Electronic Library Online (SciELO) SA, managed by the Academy of Science of South Africa (ASSAf), is a major platform for high-quality OA journals. Journals such as the *South African Journal of Science* (SAJS) operate on a diamond OA model, that is, they are free to access and free to publish, thus removing cost barriers for both readers and authors. Similarly professional associations, such as the South African Speech Language and Hearing Association (SASLHA), support their member with subsidies for APCs when publishing in the South African Journal of Communication Disorders (SAJCD).

## Why open access matters for South African researchers and clinicians in speech-language therapy and audiology

Access to research findings is fundamental for SLTs and AUDs to provide effective evidence-based care. Open access publishing addresses this need by reducing financial and legal barriers to research use. By freely sharing research articles, practice-relevant resources, intervention frameworks, implementation guides and supplementary data online, OA enables clinicians, particularly those working in resource-constrained settings, to access current, contextually relevant evidence. This, in turn, supports informed clinical decision-making and facilitates the enactment of E^3^BP.

Open access resources also empower SLTs and AUDs to deliver culturally and linguistically appropriate assessments and interventions, which are vital in multilingual countries such as South Africa by enabling access to contextually relevant research, locally developed assessment tools and evidence-based intervention approaches.

Educators and students benefit from immediate, no-cost access to up-to-date research, improving training quality across institutions, regardless of funding disparities.

In addition, OA increases the visibility and impact of South African research globally, fostering collaboration and elevating the voices of local researchers in international discourse. This democratisation of knowledge helps overcome the linguistic and structural inequalities that often marginalise researchers in LMIC.

## Barriers to research access in low- and middle-income countries

In LMICs, such as South Africa, significant barriers restrict the availability and use of research for clinicians, educators, researchers and students. These barriers limit access to contextually relevant tools and evidence, often exacerbated by paywalled publications that hinder knowledge sharing.

Barriers include a lack of resources and time to conduct effective dissemination activities, a lack of funding for APCs, limited knowledge about the availability and accessibility of culturally and contextually relevant tools, tests and interventions originating from South African-based research and a lack of skills to effectively navigate the dissemination of research results (Ellingson et al., 2021; Nieuwland et al., 2024).

Though OA publishing aims to increase research visibility, it also introduces new challenges. Open access models tend to favour studies with large datasets and quantitative methodologies, which means research in practice-based fields such as SLT and AUD, typically smaller-scale and qualitative, receives less visibility than science, technology, engineering and medicine (STEM) research (Strydom et al., 2022; Tennant et al., 2016).

Moreover, because OA journals often target wide readerships, field-specific studies in communication sciences may not be prioritised (Ajibade & Muchaonyerwa, 2023).

Another concern is the rise of predatory OA journals that exploit authors through fees without legitimate peer review (Frank et al., 2023; Ross-Hellauer, 2017). Emerging scholars are especially vulnerable to these practices, which can compromise research quality and credibility.

Furthermore, issues such as unequal internet access, digital literacy and language barriers continue to limit who benefits from OA (Piwowar et al., 2018; Shoroma & Bopape, 2023). Open access publishing does not solve these challenges; they may be partially addressed through improved research training for LMIC researchers in the fields of SLT and AUD, particularly in the context where undergraduate students receive limited research training and typically engage in research only at the end of their studies through mandatory final-year research projects.

Once qualified, most SLTs and AUDs do not continue engaging with research because of a lack of institutional support, incentives and undervaluing of research within clinical practice (Shumba & Lusambili, 2021).

Consequently, research is often perceived as a degree requirement rather than a vital component of a professional career focused on E^3^BP to ensure quality of service or ongoing clinical development.

This disconnect is evident in SLTs and AUD, where E^3^BP is acknowledged as essential, but the integration of evidence generation and use remains weak (Greenwell & Walsh, 2021). The clinician–researcher concept, which emphasises the integration of research and clinical practice whilst contributing practice-informed evidence, is difficult to implement in South Africa and other LMICs (Dakhil et al., 2024; Khoza-Shangase, 2025), where heavy clinical caseloads and workforce shortages force SLTs and AUDs to prioritise service delivery over research engagement. Nonetheless, this concept holds potential to improve healthcare processes and E^3^BP (McCurtin & O’Connor, 2020). Practice-based research acknowledges the value of understanding clinical decision-making in everyday contexts as an important complement to the evidence generated in laboratories (Crooke & Olswang, 2015). This requires clinicians to collaborate closely with researchers. Clinician–researcher collaboration requires establishing joint professional and research goals and dividing roles and responsibilities based on interpersonal communication styles and each professional’s knowledge, skills and scope of practice (Craig, 2014).

Limited access to research outputs remains a significant barrier for clinician–researchers in South Africa. In the absence of OA publishing, information about local research, resource development and conceptual adaptation efforts often remains fragmented, inaccessible or confined to institutional silos. Open access publishing offers a practical mechanism for improving disciplinary visibility and knowledge flow by making research outputs openly discoverable by clinicians, educators and researchers alike. For clinicians, this transparency supports informed decision-making by clarifying what evidence exists, where gaps remain, and when engagement with tool developers or distributors may be warranted. From an academic perspective, OA publication enables research to serve its intended clinical purpose, whilst also signalling areas of unmet need that can guide future development and innovation.

## Why open access matters for research on assessment tools and intervention strategies in South African languages

For clinicians working in the multilingual South African context, access to relevant research is not a luxury, but a prerequisite for ethical and effective E^3^PB. When research on assessment tools or intervention strategies developed for or translated into any of the 11 official spoken South African languages is published behind paywalls, its clinical value is fundamentally compromised. Open access publishing should therefore not merely be seen as an academic preference, but a mechanism through which research can meaningfully inform practice and ultimately impact society, particularly in under-resourced settings.

Much research in SLT and AUD is undertaken with the explicit aims of addressing linguistic inequities and improving services for speakers of under-represented languages (Bornman & Louw, 2021; Bornman et al., 2018). However, when the outcomes of this work are accessible only through subscription-based journals, clinicians in public health, education and community settings are effectively excluded from using the very evidence intended to support them. Open access publishing directly addresses this disconnect by enabling clinicians to engage with research findings, implementation guidance and practice-relevant insights without financial or institutional barriers.

Importantly, OA does not imply unrestricted distribution of copyrighted tools, but rather the open sharing of knowledge about assessment and intervention practices, including development processes, adaptation considerations, outcome evidence and implementation lessons. For clinicians, this transparency supports informed clinical reasoning, appropriate engagement with tool developers or distributors and context-sensitive decision-making. For academics, it ensures that research outputs fulfil their intended social and clinical purpose.

The value of OA is evident in the widespread uptake of openly accessible clinical resources (Daelmans et al., 2021), such as those developed by the World Health Organization (WHO et al., 2018), which include developmental surveillance checklists and multilingual tools like the Intelligibility in Context Scale (ICS) – Multilingual Children’s Speech, developed by McLeod et al. (2015). In South Africa, professional platforms (e.g. the SASLHA website and The Child Language Development Node (www.saslha.co.za, n.d.; https://sa-cdi.org/publications/) similarly enable clinicians to access locally relevant resources, case-based evidence and practice-informed research. These examples illustrate how OA enhances dissemination, encourages reuse and supports professional learning across diverse service contexts.

In addition to improving access, OA publishing plays a critical role in advancing the decolonisation of research and clinical practice (Mncube, 2024). By making research on South African languages visible and accessible, OA challenges exclusionary paywalled publishing models and affirms the authority of locally generated linguistic and clinical expertise as a legitimate source of scholarly knowledge. In this way, OA supports collaboration across clinicians, researchers and communities and strengthens the relevance and impact of research for practice.

The translation and development of tools, tests and interventions approaches in South Africa’s official languages is a necessary step towards equitable service delivery by SLTs and AUDs. However, without OA dissemination, the potential of this work is likely to remain unrealised. Open access can therefore be understood not simply as a publishing model, but as an ethical and practical imperative for clinicians and researchers committed to socially responsive practice.

## Addressing challenges in open access publishing

Despite its promise, OA publishing for South African researchers can be prohibitively expensive, and many researchers lack awareness of OA options or the support needed to navigate copyright and licensing frameworks. This gap partly explains why LMIC researchers often collaborate with partners in high-income countries (HICs) who may have access to funding or agreements (such as Sweden’s BIBSAM) to subsidise OA costs (Strydom et al., 2022).

To fully realise the benefits of OA, local institutions and funders must support capacity-building initiatives that educate researchers about OA publishing models and provide funding to cover APCs. Such efforts would enable more South African SLT and AUD clinician–researchers to publish and share their work widely without the financial burden of high publishing costs (Hartman & Wu, 2018; Strydom et al., 2022).

## Aligning with global trends

Encouraging South African researchers and scholars to publish through OA aligns with global efforts, mostly driven by HICs, to promote equitable knowledge sharing.

Initiatives such as the BIBSAM agreement mentioned earlier, which supports open science and responsible research assessment in Africa, reflect a growing recognition that knowledge must be freely accessible bites to drive meaningful impact. Similarly, local initiatives, such as the drive by SciELO in America, call for open science and emphasise the need for transparent, inclusive and widely accessible research to drive research uptake and impact.

If researchers publish OA, clinicians can meaningfully contribute to these initiatives. The work of these clinician–researchers, typically rooted in complex, multilingual and culturally diverse contexts, offers unique insights that are under-researched in the global discourse. When these insights remain behind paywalls, their reach is limited.

Thus, OA provides a pathway for African-based knowledge to become more visible, valued and influential. This allows SLT and AUD clinician–researchers not only to participate in global conversations, but also to contribute to and shape them, thereby ensuring that research from the continent informs international theory, policy and practice. Aligning with the OA movement is not merely a matter of compliance; it is a strategic and ethical choice that amplifies African voices and fosters more equitable scholarship.

## Practical strategies for clinicians to access research and open access publishing opportunities

Clinicians working outside academic institutions often face barriers to both publishing and accessing research. The following practical strategies are intended to support clinicians with limited institutional access in navigating these challenges:

Consider OA as a strategy to address local knowledge gaps: In South Africa and across the African continent, freely accessible evidence to support E^3^BP plays a critical role in generating contextually relevant knowledge and addressing local clinical and service-delivery challenges. Greater reliance on OA dissemination can enhance the visibility and uptake of research from the Global South, particularly in contexts where access to subscription-based journals is limited (Onaolapo et al., 2025). For clinicians, engaging with OA – both as users and contributors – offers a pathway to ensure that locally grounded evidence is accessible, reusable and responsive to community needs.

Explore OA fee waivers and discounts: Many OA journals offer full or partial APC waivers for authors based in LMICs (Abdel-Razig et al., 2024). These options are typically outlined on journal websites and can be requested during the submission process, making OA publishing more feasible for clinicians without dedicated research funding.

Engage with academic and professional partners to enhance access and visibility: Clinicians who are not formally affiliated with universities or research institutions are encouraged to seek collaborative engagement with academic researchers, professional associations or research networks. Such partnerships can enable access to institutional library resources, OA funding mechanisms and mentorship in navigating publishing systems, whilst also supporting the visibility and dissemination of clinically grounded knowledge from resource-constrained contexts (Ajibade & Muchaonyerwa, 2023).

Use alternative access pathways for non-OA publications: When research articles are behind paywalls, clinicians may access content through legal alternatives such as institutional repositories, preprint servers, author-hosted versions or by directly requesting copies from authors via email or academic networking platforms (Singh, 2023). These approaches provide practical means of accessing evidence without infringing copyright and are particularly relevant for clinicians practising outside academic institutions.

Prioritise OA-friendly journals and platforms that support collaboration and visibility: When disseminating practice-based evidence, clinicians may consider journals and platforms that support affordable OA models, self-archiving or shared dissemination practices. Selecting venues that encourage dialogue, reuse and cross-disciplinary engagement can support wider access as well as the ongoing evaluation and development of knowledge, which is particularly important in resource-constrained and practice-driven contexts (Väänänen et al., 2024).

Together, these strategies provide clinicians with feasible options for accessing and disseminating research evidence, thereby strengthening evidence-informed practice in contexts where institutional access to scholarly literature is limited.

## Call to action

The authors of this clinical perspective therefore call on clinician–researchers in SLT and AUD, practising E^3^BP, to commit, individually and collaboratively, to improving the availability and accessibility of research findings to support the development and use of resources in our field. We advocate that one method to achieve this goal is through OA publication and full use of supplementary material availability, as well as OA data storage.

The target of our advocacy must be fourfold. Firstly, we need to develop close collaborations with research managers at universities and research institutes who are tasked with securing funding and supporting researchers in disseminating research outputs for the widest possible impact of research activities.

Secondly, the target audience of our advocacy should be funding bodies. Most funding bodies will only support OA for research that they have funded, but funding for SLT and AUD-related research and research in the field of disability sciences is limited. Some funding bodies are affiliated with OA platforms that strongly advocate for OA in many different formats. However, in most cases, these funding bodies are limited to the fields within STEM and exclude disciplines such as SLT and AUD. Therefore, raising awareness is required amongst relevant funding bodies of the needs within our fields of research, potentially through science journalists and lay media channels, to alert funders to the inequity and challenges faced by researchers in these fields.

Thirdly, our allies and collaborators should be library institutes and consortia, such as BIBSAM, as they can support and initiate negotiations with publishing houses for license agreements for electronic information resources and publishing agreements (Adil et al., 2024; F1000, n.d.).

Finally, advocacy efforts should target universities, where research is taught and where research dissemination practices can be shaped to emphasise OA. In cases where relevant norms and tools are developed, these should be included in the appendices of peer-reviewed articles or supplementary materials and included in theses. Other service providers who offer Continuing Professional Development (CPD) activities could also provide relevant information about OA as the publishing landscape develops. In some instances, collaborations between international professional associations exist, allowing for shared resources. These could also be strengthened to increase the impact of collaborations. Professional associations should, in turn, advocate with the government and policymakers to release funding for the OA of all research outputs.

## Conclusion

In summary, access to relevant research is critical for advancing SLT and AUD knowledge in South Africa. Financial, infrastructural, linguistic and cultural barriers significantly limit research access for clinicians, researchers, educators and students, especially in underserved contexts and when seeking evidence related to less-resourced languages. Paywalled publications further exacerbate these challenges by restricting access to contextually appropriate research tools and findings.

Open access publishing offers a powerful means of democratising knowledge by enabling broader engagement with research evidence amongst those who need it most. By outlining OA models and available access pathways, this opinion article aims to raise awareness amongst clinician–researchers of practical opinions for accessing and disseminating scholarly work. Greater engagement with OA has the potential to strengthen clinical practice, build local research capacity and ultimately improve outcomes for the diverse communities served by South African SLTs and AUDs.
